# Our New York Letter

**Published:** 1891-09

**Authors:** 


### OUR NEW YORK LETTER.

NEW YORK, August 17, 1891.

Dr. A. J. McCosh, visiting surgeon to the Presbyterian Hospital, recently performed, at that institution, a vaginal hysterectomy for complete prolapse of the uterus on a patient forty-one years of age. The patient had been previously subjected to several minor operations for the cure of this condition, but without being afforded any material relief. After the uterus had been removed, the cervix was found to measure three and a-half inches in its long diameter, a great increase in length over the normal cervix, which measures but one inch and a-half.

This was the seventh vaginal hysterectomy performed by Dr. McCosh, six of them being for cancer of the uterus, and all recovered. Speaking of clamps and ligatures in this connection, the operator said that he used either a clamp or a ligature in accordance with the indications of each case, and he has made use of one as often as he has of the other. The form of ligature he usually employs is a twisted silk ligature of moderate size, which has been thoroughly sterilized. As far as the risk of hemorrhage goes, he considers a clamp as safe as a ligature.

He thinks the operation of vaginal hysterectomy is indicated in every case where the uterus is adherent, where the malignant disease has extended into the broad ligaments, or where the anterior wall of the bladder is affected. Under these circumstances, they would get more favorable results from vaginal hysterectomy than from high amputation of the cervix.

Injections of aniline chloride are being used by Dr. C. E. Bruce in cases of carcinoma and epithelioma at the alms house on Blackwell's Island, this city, with very satisfactory results. One patient, with epithelioma of the tongue, infiltration of the submaxillary glands, and a fixed condition of the muscles of the jaw, so as to render mastication of food impossible, was placed upon injections of ten minims of a ten per cent. solution of the aniline chloride, and within the space of three weeks the glandular infiltration subsided to such a degree that he was able to thoroughly masticate his food. The infiltration surrounding the epithelioma was diminished to such an extent that the deglutition of solid food was rendered not only possible but even comfortable.

Another case was one of carcinoma of the uterus, where the infiltration of the uterine tissue was so great, and the os so swollen, that it was hardly possible to get it within the opening of the bivalve speculum. Within the brief space of a month, under this treatment, the induration had been reduced to the size of a silver dollar; the general condition of the patient was good; she had increased in flesh and strength, and experienced no further pain or discomfort. Previous to going under treatment, she had been in the habit of passing great quantities of blood from the vagina, but this has now given place to a thin, watery, colorless discharge. She now feels very comfortable, and is relieved from all her distressing symptoms.

Dr. Johnathan Wright, of Brooklyn, in a paper read before the Kings County Medical Association, recommends the use of thymol as a spray in the treatment of atrophic rhinitis. He has made use of the following formula with good success:

℞ Thymol, grs. jss.

Alcohol, } aa ʒ jss.

Glycerini, }

Aquae dist. ad ℥ j.

The advantages he claims for thymol are: it is a good antiseptic; it has a pleasant odor; it is sufficiently soluble for sprays and douches; it stimulates the mucous membrane and causes a profuse watery secretion; it is not poisonous, and has no constitutional effect; it produces no vascular constringency. This process of cleansing the nasal fossae and stimulating of the mucous membrane must be repeated every other day for many months, and then, when it is found that the patient is able to keep the nasal cavities clean in the intervals at home, these intervals may be lengthened gradually to once or twice a week, or once in two weeks.

It is necessary that the patient should use a cleansing solution at home. Siler's tablets, or the Dobell solution may be used with syringe, spray or douche-cup. When the patient is able to go for a week or ten days without help in cleansing her nose, and without a return of the odor or other symptoms, we may conclude that some permanent benefit has been attained. The case should not be dismissed, however, until the mucous membrane is seen to be constantly moist, pink and free from dried secretions.

Dr. Weir, clinical professor of surgery at the College of Physicians, this city, speaking at a recent clinic of the therapeutic value of pyoktannin in cancer, expressed himself in the following manner:

"I wish in this connection to say a few words with regard to the new remedy for the cure of neoplasms, and which is known by the name of "pyoktannin," as you may be interested to know its therapeutic effects at the New York Hospital. I have made as many as thirty or forty injections with this remedy in cases of cancer of the neck, and without the slightest improvement being noted. In a case of cancerous growth of the upper jaw, it had apparently produced some temporary benefit, causing in certain portions of the mass hardness and a general shrinkage of tissue.

"A patient came to my office a few days ago on whom I used this remedy with some benefit. Three years ago I operated on a private patient with epithelioma of the tongue, removing with the knife the whole of the diseased structure. He was at that time an inveterate smoker, and I advised him to abandon the use of tobacco altogether. He did so, and came to my office at a later period asking if he could again take up his cigar. I told him he might do so moderately, but he did not pay any regard to the meaning of the word "moderately," and began to smoke at the rate of five or six cigars a day. He soon came back with a whitish film on that side of his tongue from which the epithelioma had been previously removed. The affected tissues were in that state known by the name of "ichthyosis." I am always suspicious of any cancerous trouble presenting under such circumstances as this. Many surgeons regard it as a precancerous stage of the disease. I recommended that he should stop smoking altogether, and advised the use of a solution of pyoktannin in the proportion of 1-300. After five or six applications of the lotion the trouble disappeared altogether. This only proves, if it proves anything for this drug, that its application where there is a tendency to cancer, or changes in the mucous membrane, is of some benefit in arresting the development of a neoplasm."

